# Biphasic tissue expression of cfa-miR-409-3p and cfa-miR-4270 during malignant transformation in canine mammary tumors: an exploratory study

**DOI:** 10.3389/fvets.2026.1861662

**Published:** 2026-06-23

**Authors:** Luíz Guilherme Dércore Benevenuto, Zara Alves Lacerda, Reiner Silveira de Moraes, Patricia de Faria Lainetti, Ana Giulia Gabriel da Rocha Cesario, Alexandre Matheus Baesso Cavalca, Renee Laufer Amorim, Carlos Eduardo Fonseca-Alves

**Affiliations:** 1 Department of Veterinary Surgery and Anesthesiology, Faculty of Veterinary Medicine and Animal Science, São Paulo State University (UNESP), Botucatu, Brazil; 2 Department of Veterinary Clinics, Faculty of Veterinary Medicine and Animal Science, São Paulo State University (UNESP), Botucatu, Brazil

**Keywords:** adenoma-to-carcinoma transition, biomarkers, canine mammary tumors, comparative oncology, liquid biopsy, microRNA, RT-qPCR

## Abstract

**Introduction:**

Canine mammary tumors (CMTs) are the most common malignancy in intact female dogs and represent a well-established spontaneous model for human breast cancer. MicroRNAs (miRNAs) are post-transcriptional regulators of gene expression critically involved in carcinogenesis and are candidate non-invasive biomarkers for tumor diagnosis and prognosis.

**Methods:**

This exploratory study evaluated the tissue and plasma expression of five candidate miRNAs—cfa-miR-133a, cfa-miR-127-3p, cfa-miR-652, cfa-miR-409-3p, and cfa-miR-4270—in two independent cohorts. The tissue cohort comprised 57 mammary samples (33 carcinomas, 14 adenomas, and 10 normal tissues) analyzed by poly(A) tailing-based SYBR Green RT-qPCR. The plasma cohort included 75 dogs with mammary carcinomas and 5 healthy controls, quantified by TaqMan Advanced miRNA Assays.

**Results:**

Tissue analysis revealed that cfa-miR-133a was significantly downregulated in both adenomas and carcinomas relative to normal tissue (*p* < 0.001), consistent with early and sustained tumor suppressor loss during mammary carcinogenesis. Notably, cfa-miR-409-3p and cfa-miR-4270 displayed a biphasic expression pattern: significantly downregulated in adenomas relative to normal tissue and partially restored in carcinomas—a biphasic pattern that was statistically significant for cfa-miR-4270 (*p* = 0.008) and showed a consistent trend for cfa-miR-409-3p (*p* = 0.054)—suggesting context-dependent regulation during the adenoma-to-carcinoma transition. In plasma, no statistically significant differences were detected across all clinical comparisons, including carcinoma vs. healthy controls, metastatic vs. non-metastatic disease, tumor stage, reproductive status, and survival outcomes.

**Discussion:**

These findings indicate that tissue-level miRNA dysregulation in CMTs follows biologically coherent, progression-associated patterns aligned with the roles of human orthologs in breast cancer, whereas no significant circulating signatures were detected in plasma; however, the plasma analyses were limited by a small control group and were underpowered, and these negative findings should not be interpreted as evidence against biological relevance. These results support the design of larger, prospectively standardized investigations to validate tissue miRNA biomarkers and advance liquid biopsy approaches in canine mammary oncology.

## Introduction

Canine mammary tumors (CMTs) are the most common neoplasms in intact female dogs, accounting for approximately 50% of all tumors diagnosed in this population (1). Their pathological spectrum—ranging from benign mixed tumors and adenomas through to invasive carcinomas and metastatic disease—closely parallels the histological and molecular diversity observed in human breast cancer (2). This biological analogy, reinforced by shared epidemiological risk factors including reproductive status and hormonal exposure, has established dogs as a valuable large-animal model for translational breast cancer research (3). Comparative genomic and transcriptomic analyses have demonstrated conservation of key oncogenic pathways between CMTs and human breast cancer, including PI3K/AKT/mTOR, MAPK, Wnt/*β*-catenin, and JAK–STAT signaling (4). The spontaneous nature of CMT development—occurring without artificial carcinogen exposure—further enhances the relevance of this model for biomarker discovery and evaluation of early detection strategies.

MicroRNAs (miRNAs) are small non-coding RNAs of approximately 17–24 nucleotides that post-transcriptionally regulate gene expression by binding to complementary sequences in the 3′ untranslated regions (3′UTR) of target mRNAs, leading to mRNA degradation or translational repression (5). Dysregulation of miRNA networks is a hallmark feature of virtually all human malignancies (6), with individual miRNAs functioning as oncomiRs or tumor suppressors depending on cellular context, target gene availability, and tumor microenvironment cues. In human breast cancer, miRNA profiling has identified numerous diagnostic and prognostic candidates (7, 8). In CMTs, parallel studies have characterized differentially expressed miRNA landscapes using microarray and next-generation sequencing platforms (1, 9–12), establishing a foundation for targeted validation studies using sensitive quantitative methods.

Translating tissue-based miRNA signatures into circulating liquid biopsy assays represents a major goal in oncology, as plasma miRNAs offer the advantages of non-invasive sampling, serial monitoring, and reduced tissue heterogeneity bias (13, 14). However, clinical translation has been hampered by pre-analytical variability in plasma collection, instability of commonly used reference genes in biofluids, and insufficient evidence that tissue-level dysregulation is reflected in detectable plasma concentration changes (4, 15). These challenges are particularly pronounced in veterinary medicine, where standardized protocols for circulating miRNA quantification remain under development (9). Understanding the relationship between tissue and plasma miRNA levels in CMTs is therefore essential to inform the feasibility of non-invasive biomarker strategies in canine oncology.

In this exploratory study, we evaluated the tissue and plasma expression of five candidate miRNAs—cfa-miR-133a, cfa-miR-127-3p, cfa-miR-652, cfa-miR-409-3p, and cfa-miR-4270—selected based on their dysregulation in prior CMT profiling studies and on the documented roles of their human orthologs in mammary and other cancers. Tissue expression was assessed across the adenoma-to-carcinoma sequence, enabling evaluation of miRNA dynamics during progressive malignant transformation. Plasma levels were measured in a separate cohort of dogs with mammary carcinomas and healthy controls to evaluate liquid biopsy feasibility. We hypothesized that tissue miRNA expression would follow biologically coherent, progression-associated patterns, and that tissue-level changes would be reflected in detectable plasma differences.

## Methods

### miRNA selection

Candidate microRNAs (cfa-miR-133a, cfa-miR-127-3p, cfa-miR-652, cfa-miR-409-3p, and cfa-miR-4270) were selected through a structured literature search of human breast cancer and canine mammary tumor studies. Selection criteria included: (1) evidence of differential expression in mammary tissue or blood in at least one published study; (2) biological plausibility based on known target pathways; and (3) availability of validated or in silico-predicted canine orthologues in miRBase. None of the five candidates had been previously validated as circulating biomarkers in dogs at the time of study design, representing the gap this work aimed to address. Details are summarized in Supplementary Table S1.

### Study population and sample collection

**Figure 1:**
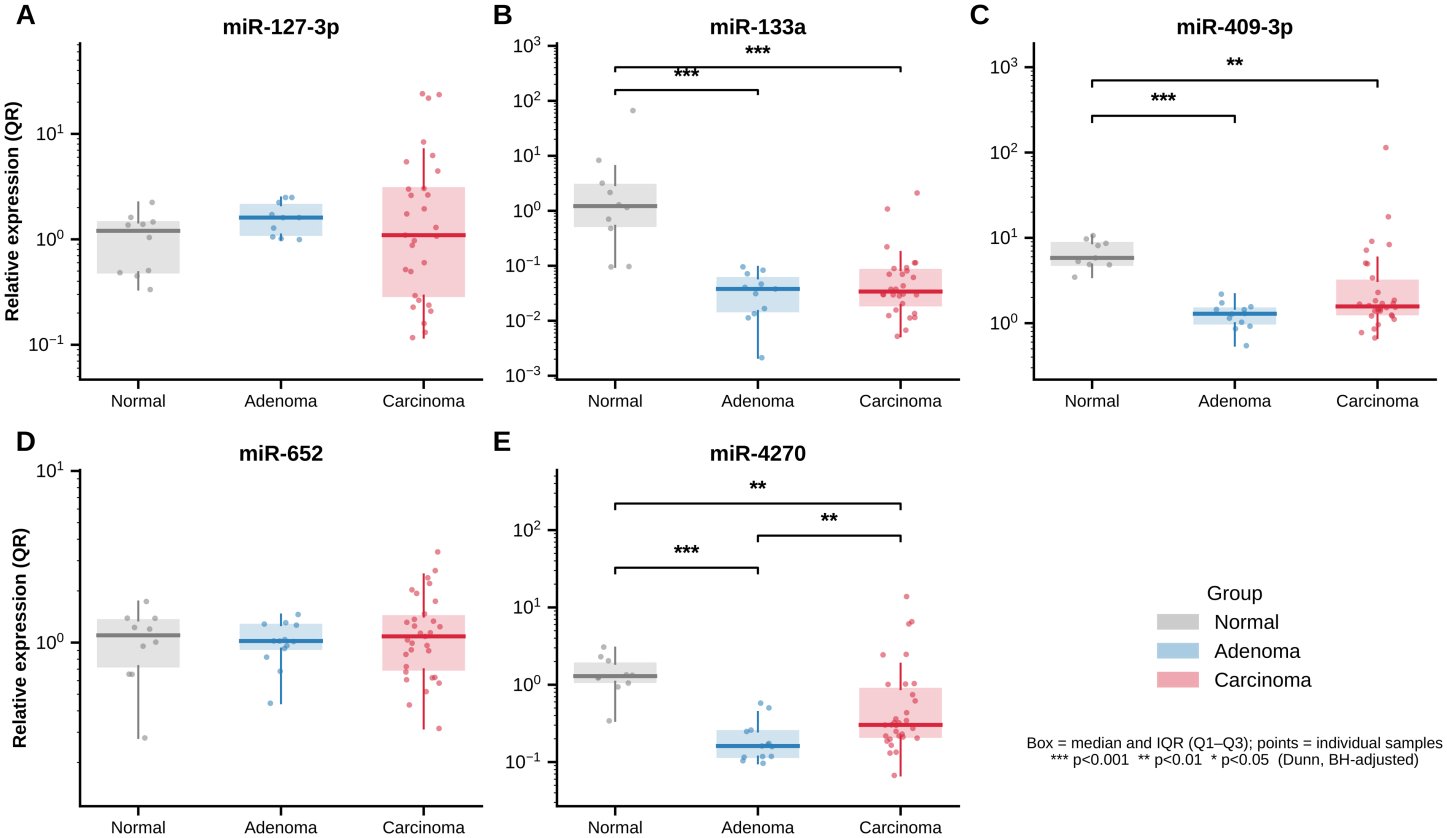
Tissue expression of candidate microRNAs across the canine mammary adenoma-to-carcinoma sequence. Relative tissue expression of candidate microRNAs in canine mammary tissues (normal, adenoma, carcinoma). Boxes represent the median and interquartile range (IQR); individual points are single samples and the y-axis is on a log scale. Panels **(A–E)** display cfa-miR-127-3p, cfa-miR-133a, cfa-miR-409-3p, cfa-miR-652 and cfa-miR-4270 across the three tissue groups (Kruskal–Wallis test followed by Dunn’s multiple-comparison test with Benjamini–Hochberg adjustment). Asterisks denote statistically significant pairwise comparisons (*p < 0.05; **p < 0.01; ***p < 0.001); ns, not significant.

Two independent cohorts were evaluated. The tissue cohort comprised 57 archival formalin-fixed, paraffin-embedded (FFPE) mammary samples, including 33 carcinomas, 14 adenomas and 10 histologically normal mammary tissues; these tissues had been collected from dogs undergoing mastectomy between August 2021 and November 2023. Complete clinicopathological data (age, histological grade and clinical stage) were available for a subset of cases and are summarised in Table 1, whereas microRNA expression was quantified in all tissue samples (Figure 1). The plasma cohort was prospectively designed and included 75 female dogs with mammary carcinomas and 5 clinically healthy controls (Supplementary Figure S1). All procedures were approved by the Institutional Animal Care and Use Committee (Protocol No. CEUA 0227/2021). The normal mammary tissues were obtained at necropsy from dogs that died from causes unrelated to mammary disease and showed no clinical or pathological evidence of mammary neoplasia. No animal received anticancer treatment (chemotherapy or hormonal therapy) prior to sample collection. Clinical follow-up data were available only for the prospective plasma cohort; the archival FFPE tissue cohort did not have associated longitudinal follow-up.

**Table 1:** Clinical parameters of dogs in the carcinoma and ademona groups.

Clinical parameters	*n*	%
Carcinoma group		
Age (years)		
<10	9	52.94
>10	8	47.06
Reproductive status		
Intact	11	64.71
Spayed	6	35.29
Tumor size		
<5 cm	7	41.18
>5 cm	10	58.82
Histological grade		
I	9	52.94
II	6	35.29
III	2	11.76
Staging		
I	3	17.65
II	5	29.41
III	9	52.94
IV	0	0
V	0	0
Adenoma group		
Age (years)		
<10	11	91.67
>10	1	8.33
Reproductive status		
Intact	7	58.33
Spayed	5	41.66
Tumor size		
<5 cm	9	75
>5 cm	3	25

Evaluated variables include age (<10 years and >10 years), neutering status (intact and neutered), tumor size (<5 cm and >5 cm), histological grade (grades I to III for carcinoma), and staging (stages I to V for carcinoma). Data are presented as the number of individuals (*n*) and percentages (%).

Plasma samples were collected prior to surgery into EDTA-containing tubes and processed immediately after collection. Blood was centrifuged at 4,000 rpm (≈2,000 × *g*) for 15 min at 4 °C, and the resulting plasma was re-centrifuged at 1,600 × *g* for 10 min at 4 °C to remove residual cellular debris before storage at −80 °C in aliquots. Hemolysis was assessed visually; grossly hemolyzed samples were excluded (a limitation discussed below). Tumors were classified histologically according to the WHO classification system for canine mammary tumors [(19), Vet Pathol] and graded using the Elston-Ellis system adapted for canine mammary neoplasms. All histopathological diagnoses were performed or reviewed by board-certified veterinary pathologists (R.L.A. and C.E.F.A.). There was no overlap between normal tissues and plasma controls.

### RNA extraction and cDNA synthesis

RNA was extracted from 10-μm FFPE sections, with tumour-rich areas macrodissected under the supervision of a board-certified pathologist, using the miRNeasy FFPE Kit (QIAGEN). Plasma RNA was isolated using the miRNeasy Serum/Plasma Advanced Kit (QIAGEN). RNA was stored at −80 °C. RNA quality and yield were assessed on a NanoDrop 2000 spectrophotometer (Thermo Fisher Scientific); FFPE tissue samples with A260/280 ratio between 1.8 and 2.1 were considered acceptable, and plasma samples with insufficient yield were excluded.

Prior to cDNA synthesis, tissue RNA was subjected to poly(A) tailing using poly(A) polymerase to generate 3′ poly(A) extensions on mature miRNAs, enabling efficient capture by oligo(dT)-based priming. cDNA synthesis was subsequently performed using SuperScript III reverse transcriptase (Thermo Fisher Scientific) with a poly(T) adapter primer containing a 3′ anchor sequence. This poly(A) tailing approach enables specific and efficient reverse transcription of mature miRNAs of 17–24 nucleotides and has been previously validated for SYBR Green-based miRNA quantification. Plasma miRNA cDNA was synthesised using the TaqMan Advanced miRNA cDNA Synthesis Kit. Negative controls without reverse transcriptase were included.

### RT-qPCR analysis

MicroRNA expression was quantified by RT-qPCR on a StepOnePlus Real-Time PCR System (Applied Biosystems) using SYBR Green chemistry. Each 10 μL reaction contained SYBR Green Master Mix, specific forward and universal reverse primers, and template cDNA. Cycling conditions consisted of 95 °C for 10 min, followed by 40 cycles at 95 °C for 15 s and 60 °C for 60 s. All reactions were run in technical triplicate on the same plate, with the five candidate microRNAs and the U6 snRNA endogenous reference assayed together for each sample to minimise inter-assay variability. U6 snRNA has previously been employed as a reference gene for microRNA quantification in both tissue and plasma (16). Melt-curve analysis was performed after each run to confirm amplicon specificity and exclude primer-dimer formation. Primer efficiency was assessed by serial dilution standard curves; assays with efficiency outside 90%–110% (*R*^2^ > = 0.99) were excluded. Inter-run variability was controlled by including an inter-plate calibrator sample across all runs. Expression data were analysed by the 2^−ΔΔCt^ method relative to U6. Primer sequences for all candidates are listed in Supplementary Table S2.

### Prospective clinical study design for plasma microRNA analysis

This prospective, observational study included female dogs affected by mammary gland tumours that underwent surgical procedures. All procedures were pre-approved by the Institutional Animal Care and Use Committee under Protocol No. CEUA 0227/2021.

Prior to the procedure, all dogs underwent a standard clinical examination, which included a medical history review, a comprehensive physical examination, documentation of tumor characteristics, hematological analysis, serum biochemical profiling, and three thoracic radiographs (right and left lateral, and dorsoventral views).

Dogs presenting with conditions or pathological stages that could potentially influence the study outcomes were excluded. The patients were monitored from the time of definitive diagnosis (day 0) for a minimum follow-up period of 2 years.

### Bioinformatic analysis

Predicted target genes for the selected miRNAs were identified using the mirWalk platform (version 3.0; http://mirwalk.umm.uni-heidelberg.de), integrating predictions from TargetScan, miRDB, and miRTarBase; only targets predicted by at least two algorithms were retained. Functional enrichment was performed with DAVID (v2025_1; https://davidbioinformatics.nih.gov), applying FDR < 0.05. Signaling pathways were mapped using the KEGG database. Interaction networks were visualized in Cytoscape (version 3.10.4). All databases were accessed in February 2025.

### Statistical analysis

All statistical analyses were performed with GraphPad Prism v8.0 (GraphPad Software, San Diego, CA, USA). Normality was assessed with the Shapiro–Wilk test; because most variables departed from normality, between-group differences were evaluated with the Kruskal–Wallis test followed by Dunn’s post-hoc test (Benjamini–Hochberg-adjusted) or the Mann–Whitney *U* test, as appropriate. Data are presented as median and interquartile range (IQR). Effect sizes (epsilon-squared for Kruskal–Wallis; rank-biserial r for Mann–Whitney) and log2 fold-changes with bootstrap 95% confidence intervals are reported (Supplementary Table S3). Given the exploratory, hypothesis-generating nature of this pilot study, *p*-values were not adjusted for multiple comparisons across the five candidate microRNAs; nonetheless, Benjamini–Hochberg false-discovery-rate q-values are provided for transparency, and the three significant tissue microRNAs remained significant after adjustment. Survival analyses (disease-free interval and overall survival) used Kaplan–Meier curves, with dogs dichotomised at the median expression and compared by the log-rank test. Correlations were assessed with Spearman’s rank correlation. A two-sided *p* < 0.05 was considered statistically significant. Median-based dichotomization was pre-specified as a methodologically conservative, data-independent threshold; alternative cutoff strategies (e.g., maximally selected log-rank statistics or Youden-index-derived cutpoints) were considered but were not adopted because the small number of survival events in this exploratory cohort would have favored overfitting and inflated false-discovery rates.

## Results

### Demographic data from the tissue pilot study

This study provides a descriptive analysis of clinical parameters in female dogs diagnosed with mammary tumors, focusing on age, reproductive status, tumor size, histological grade, and disease stage for carcinoma and adenoma groups. Complete clinicopathological data were available for a subset of the tissue cohort (17 carcinomas and 12 adenomas; Table 1), whereas microRNA expression was quantified in all tissue samples (Figure 1).

In the carcinoma group, dogs were categorized into two age groups: under 10 years and over 10 years. The distribution was relatively balanced, with 52.94% (*n* = 9) under 10 years and 47.06% (*n* = 8) over 10 years. Regarding reproductive status, 64.71% (*n* = 11) were intact, while 35.29% (*n* = 6) were spayed, which may be relevant for understanding reproductive influences on mammary tumor development.

Tumors were classified by size, with 41.18% (*n* = 7) measuring less than 5 cm in diameter and 58.82% (*n* = 10) exceeding 5 cm. Histological grade analysis revealed that 52.94% (*n* = 9) were grade I, 35.29% (*n* = 6) were grade II, and 11.76% (*n* = 2) were grade III, indicating varying levels of tumor aggressiveness.

Disease staging ranged from I to V, with most cases classified as stage III (52.94%, *n* = 9), followed by stage II (29.41%, *n* = 5) and stage I (17.65%, *n* = 3). No cases were recorded at stages IV or V.

In the adenoma group, 91.67% of the dogs were under 10 years old, while 8.33% were over 10 years, suggesting that adenomas may be more common or more frequently diagnosed in younger dogs. Regarding reproductive status, 58.33% were intact, and 41.66% were spayed, indicating a potential association between intact status and adenoma development. Tumor size analysis showed that 75% of adenomas were smaller than 5 cm, while 25% were larger.

Table 1 summarizes these clinical parameters, providing a basis for further exploration of potential associations between these variables and disease progression or prognosis.

### microRNA tissue expression

The relative expression analysis of microRNAs cfa-miR-409-3p, cfa-miR-4270, cfa-miR-133a, and cfa-miR-127 (Figure 1) revealed significant differences among the evaluated groups.

The expression of cfa-miR-409-3p (Figure 1C) was significantly reduced in the Adenoma group compared with the Control group (*p* < 0.001). Expression in the Carcinoma group was higher than in the Adenoma group, although this increase represented a non-significant trend (*p* = 0.054); together with the significant downregulation in adenomas, this is consistent with a biphasic, stage-dependent pattern.

Similarly, cfa-miR-4270 (Figure 1E) exhibited a significant reduction in the Adenoma group compared to the Control group (*p* < 0.001). Its expression was significantly higher in the Carcinoma group than in the Adenoma group (*p* = 0.008), confirming a biphasic pattern and indicating involvement in the transition to malignancy.

In contrast, cfa-miR-133a (Figure 1B) showed significantly higher expression in the Control group compared to both the Adenoma and Carcinoma groups (*p* < 0.001), with no significant differences between the latter two groups. This suggests that downregulation of cfa-miR-133a may be more closely associated with tumor presence than with progression to malignancy.

The expression of cfa-miR-127-3p (Figure 1A) demonstrated a progressive increase across groups, with the highest levels in the Carcinoma group compared to the Control and Adenoma groups, although without apparent statistical significance.

No significant differences were observed in the analysis of cfa-miR-652 (Figure 1D).

These findings suggest that the evaluated microRNAs play distinct roles in canine mammary carcinogenesis. Specifically, cfa-miR-409-3p and cfa-miR-4270 may contribute to tumor progression, while the downregulation of cfa-miR-133a appears to be associated with the presence of neoplasia, regardless of its malignancy potential. Meanwhile, cfa-miR-127-3p may be related to tumor progression, although its biological significance requires further investigation.

### Correlation analysis

**Figure 2:**
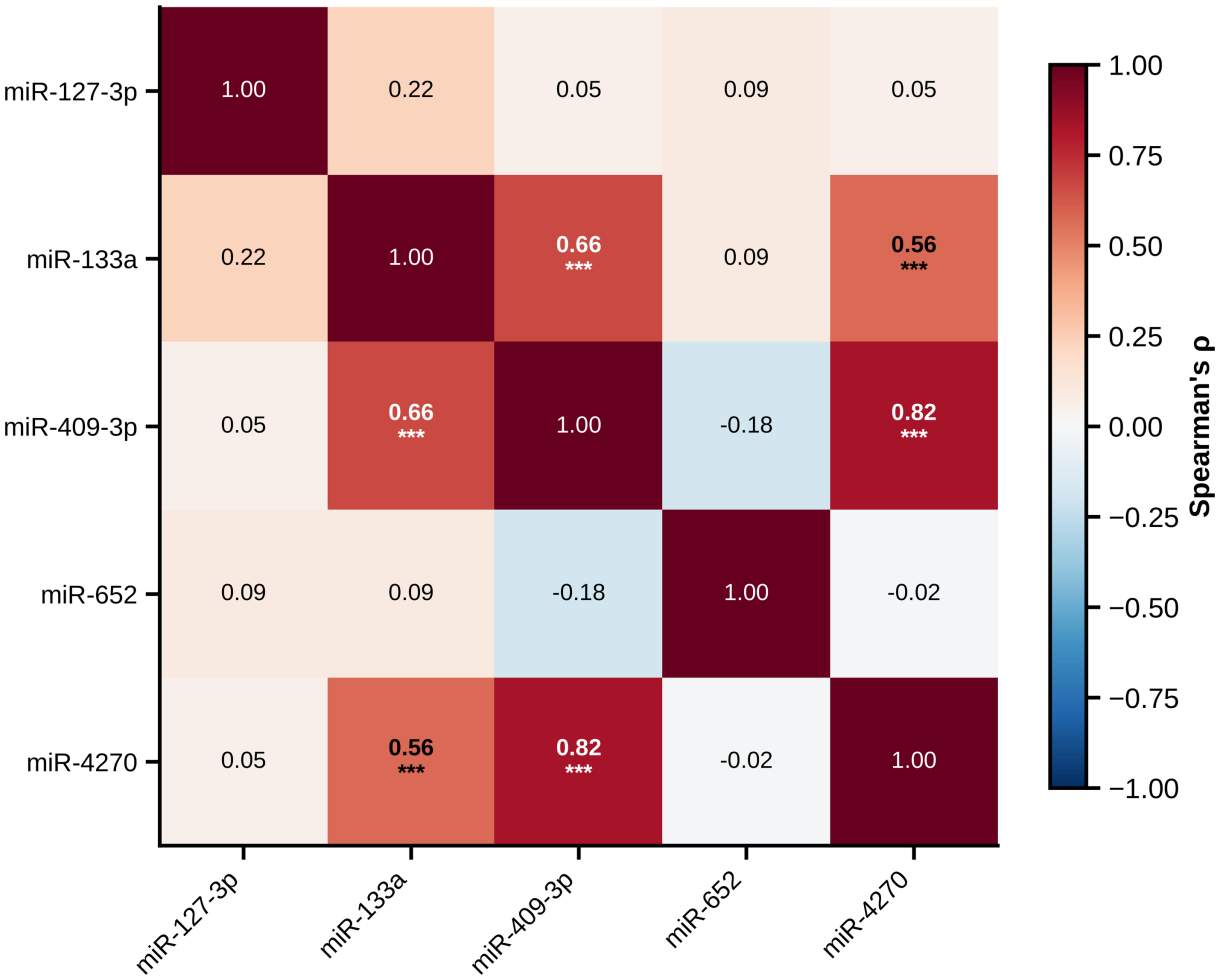
Inter-microRNA spearman correlation in mammary tissue. Spearman correlation matrix among the five candidate microRNAs measured in mammary tissue. Colour intensity indicates the strength and direction of the correlation (red, positive; blue, negative). Asterisks denote statistical significance (*p < 0.05; **p < 0.01; ***p < 0.001).

Pairwise correlation analysis among the candidate microRNAs in tissue revealed strong, statistically significant positive correlations between the three dysregulated microRNAs (Figure 2). The strongest association was observed between cfa-miR-409-3p and cfa-miR-4270 (Spearman’s *ρ* = 0.82, *p* < 0.001), consistent with their coordinate biphasic expression across the adenoma-to-carcinoma sequence. Cfa-miR-133a was also positively correlated with both cfa-miR-409-3p (*ρ* = 0.66, *p* < 0.001) and cfa-miR-4270 (*ρ* = 0.56, *p* < 0.001), whereas cfa-miR-127-3p and cfa-miR-652 showed no significant correlation with the other microRNAs.

**Figure 3:**
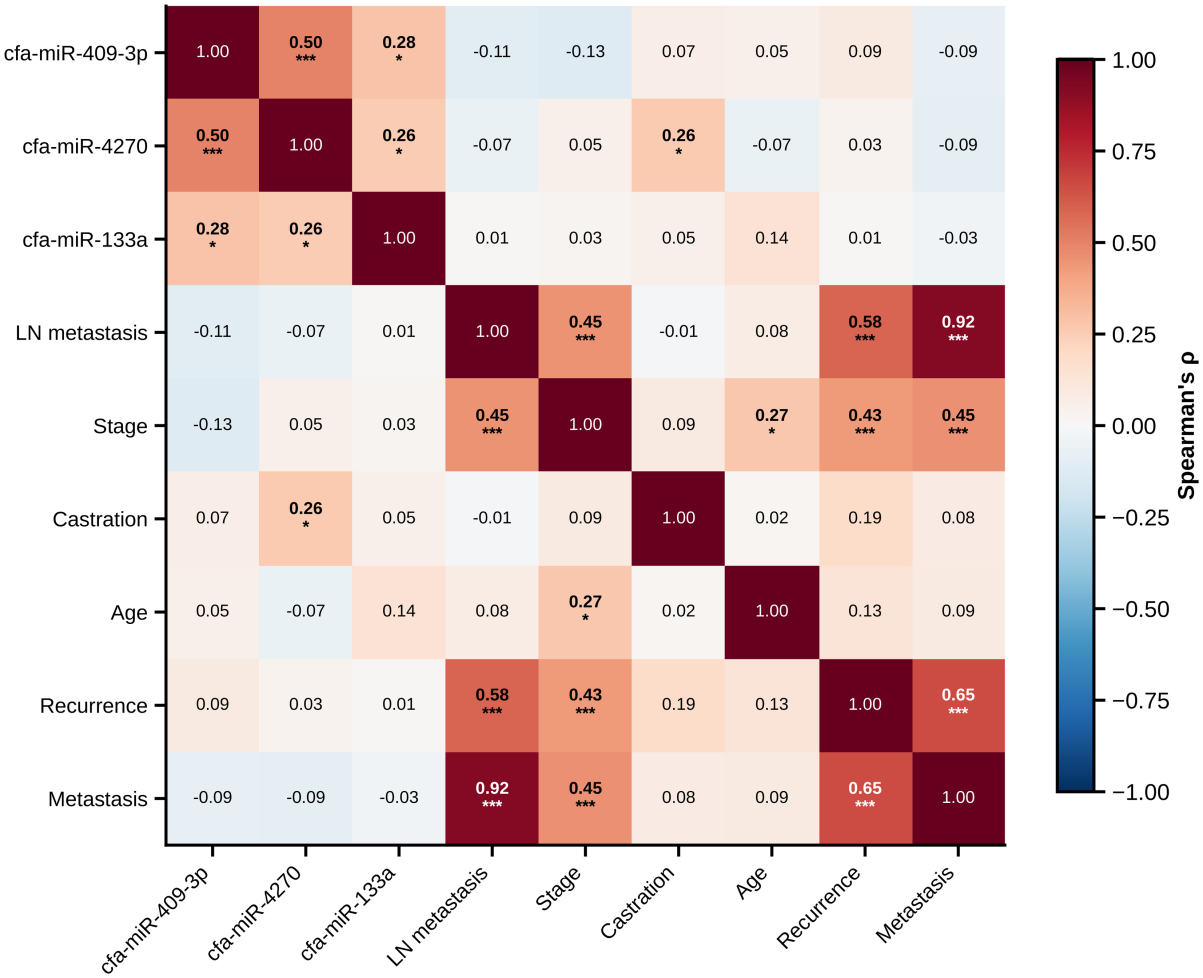
Spearman correlation matrix—plasma microRANs and clinicopathological variables. Correlation analysis between microRNA expression and clinical characteristics in female dogs with mammary tumors. The heatmap illustrates the correlation between microRNAs (cfa-miR-409-3p, cfa-miR-4270, and cfa-miR-133a) and clinical variables, including lymph node metastasis, tumor staging, age, tumor grade, and spaying status. Colors represent the strength and direction of the correlation.

Clinicopathological correlations were not evaluated in the tissue cohort, because complete clinical data were available only for a subset of cases; correlations between microRNA expression and clinicopathological variables are instead presented for the prospectively characterized plasma cohort (Figure 3).

Taken together, the strong co-expression of cfa-miR-133a, cfa-miR-409-3p and cfa-miR-4270 in mammary tissue supports the hypothesis of shared or coordinated regulatory mechanisms operating during malignant transformation, and warrants mechanistic investigation in functional models.

### Demographic data from the plasma study

The plasma cohort included 75 dogs with mammary carcinomas and 5 healthy controls. Demographic and clinicopathological data are shown in Table 2. In this group of female dogs diagnosed with mammary tumors (*n* = 75), the clinical parameter analysis revealed that most dogs were over 10 years old, accounting for 57.3% of the sample (*n* = 43/75), while 42.6% (*n* = 32/75) were younger than 10 years. Regarding reproductive status, 61.3% (*n* = 46/75) were intact, and 38.6% (*n* = 29/75) were spayed.

**Table 2:** Clinical parameters of female dogs diagnosed with mammary tumors.

Clinical parameters	*n*	%
Age (years)		
<10	32	42.6%
>10	43	57.3%
Reproductive status		
Intact	46	61.3%
Spayed	29	38.6%
Tumor size		
<5 cm	51	68%
>5 cm	24	32%
Histological grade		
I	47	62.7%
II	12	16.0%
III	4	5.3%
Staging		
I	29	38.6%
II	17	22.6%
III	17	22.6%
IV	9	12%
V	3	4%

Data includes the distribution by age, reproductive status, tumor size, histological grade, and clinical staging. Values are presented as number of cases (*n*) and corresponding percentages (%).

Tumor size analysis showed that the majority were smaller than 5 cm, representing 68% of cases (*n* = 51/75). Larger tumors (>5 cm) were observed in 32% of the dogs (*n* = 24/75). Regarding histological grade, 62.7% (*n* = 47/75) of the dogs had grade I tumors, 16.0% (*n* = 12/75) had grade II tumors, and 5.3% (*n* = 4/75) had grade III tumors.

The clinical stage distribution showed that 38.6% (*n* = 29/75) of the dogs were at stage I. Stages II and III each represented 22.6% of the cases (*n* = 17/75). More advanced stages were less common, with 12% (*n* = 9/75) of the dogs at stage IV and 4% (*n* = 3/75) at stage V.

These findings, presented in Table 2, indicate that most dogs in the study were older and unspayed, with predominantly smaller and low-grade tumors. Early-stage disease was more frequent; however, a considerable proportion of cases were found at advanced stages, highlighting the variability in the clinical presentation of canine mammary tumors.

### Plasma microRNA expression

**Figure 4:**
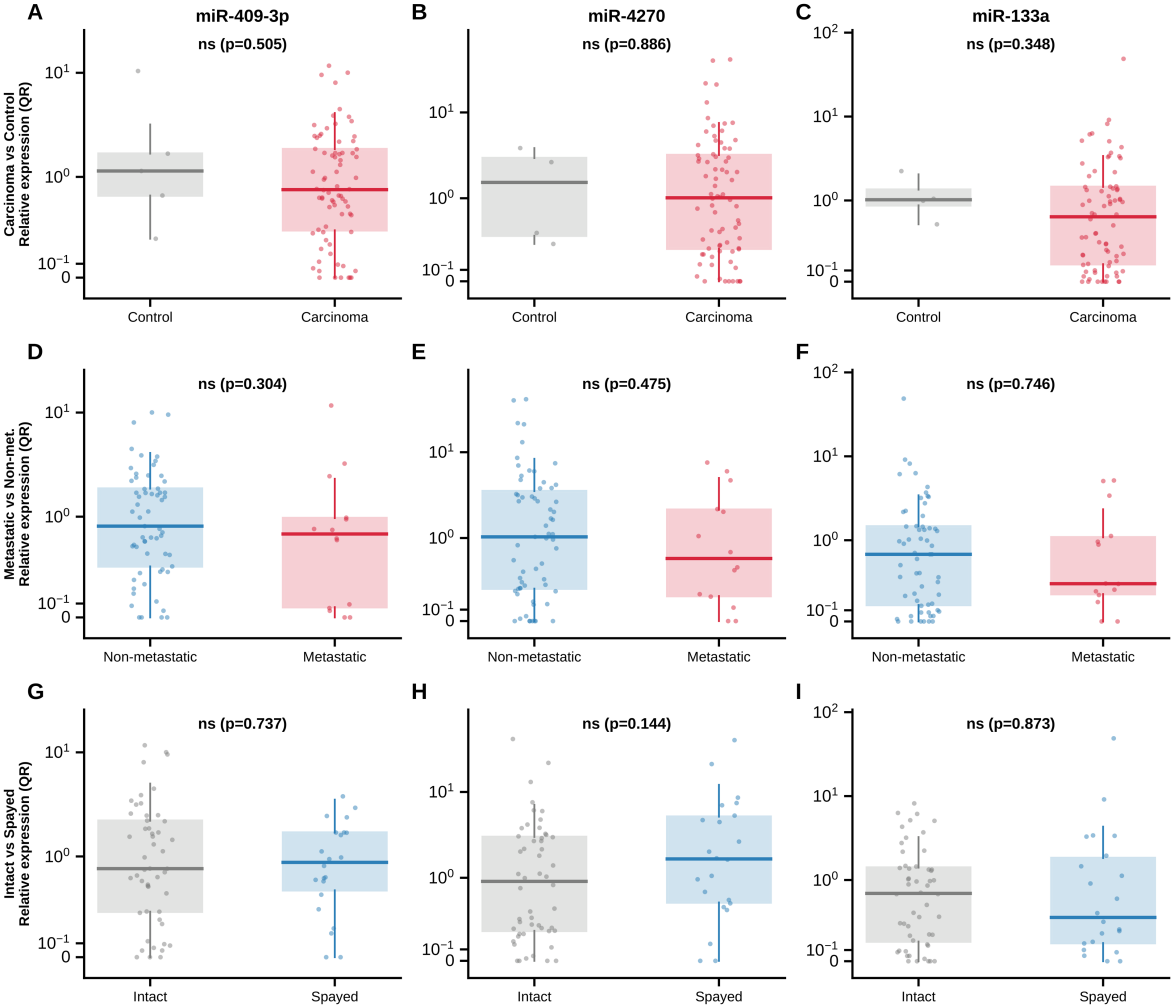
Plasma microRNA expression across clinical comparisons (all non-significant, Mann–Whitney). Relative expression of microRNAs cfa-miR-409-3p **(A,D,G)**, cfa-miR-4270 **(B,E,H)**, and cfa-miR-133a **(C,F,I)** in female dogs with mammary tumors. Expression levels were compared between the control group (healthy dogs) and the carcinoma group (dogs with malignant mammary tumors) **(A–C)**, between metastatic and non-metastatic tumors **(D–F)**, and between intact and spayed dogs **(G–I)**. No statistically significant differences were observed among groups (Mann–Whitney *U* test, *p* > 0.05). Boxes represent the median and interquartile range; individual points are single samples; ns, not significant. The y-axis is shown on a symmetric-log (symlog) scale to display non-detected samples (QR = 0) together with the full expression range.

Plasma expression of cfa-miR-409-3p, cfa-miR-4270, and cfa-miR-133a was evaluated across three clinical comparisons: mammary carcinoma versus clinically healthy controls, metastatic versus non-metastatic disease, and spayed versus intact animals (Figure 4). None of the three miRNAs differed significantly between groups in any of these comparisons. A numerical trend toward higher cfa-miR-409-3p expression was observed in intact animals, while cfa-miR-4270 and cfa-miR-133a showed slightly elevated values in the spayed group; however, none of these differences reached statistical significance. No detectable circulating signal was associated with carcinoma status, metastatic burden, or reproductive condition in this cohort; however, given the small number of healthy controls, these comparisons were underpowered, and these negative findings should not be interpreted as evidence against biological relevance.

### microRNA plasma expression and stages

**Figure 5:**
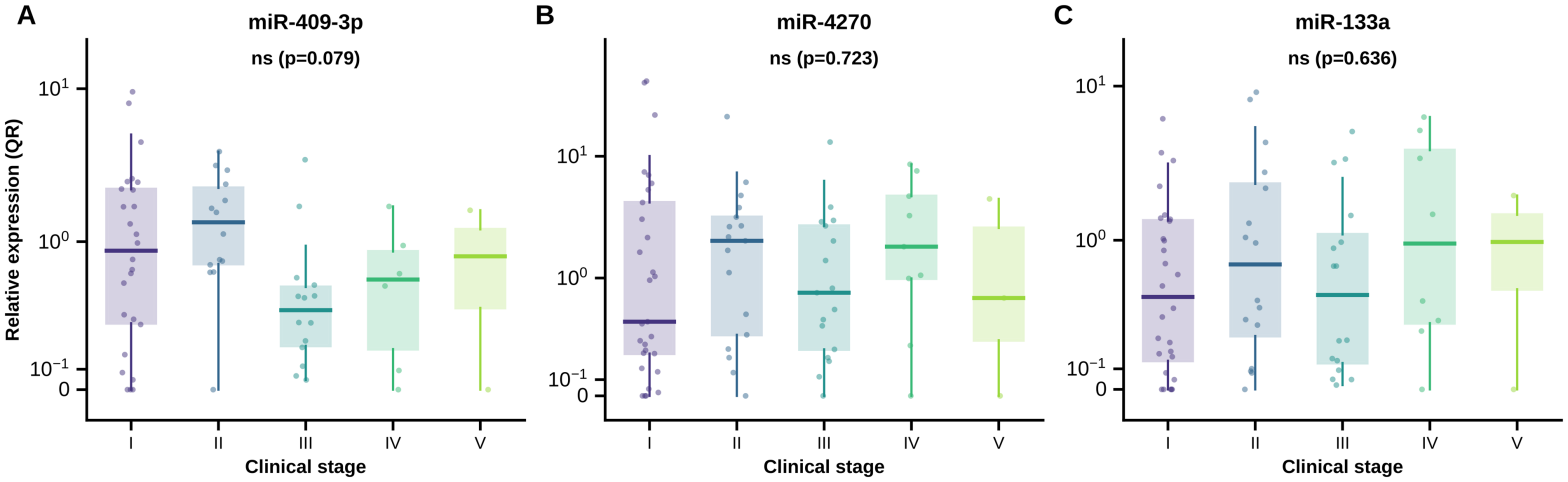
Plasma microRNA expression by clinical stage (Kruskal-Wallis, all non-significant). Relative plasma expression levels of candidate microRNAs: cfa-miR-409-3p **(A)**, cfa-miR-4270 **(B)**, and cfa-miR-133a **(C)** according ot clinical stage (I-V). Boxes represent the median and interquartile range; individual points are single samples. No statistically significant differences were observed among stages (Kruskal–Wallis test, *p* > 0.05). The y-axis is shown on a symmetric-log (symlog) scale to display non-detected samples (QR = 0) together with the full expression range.

Stage-stratified plasma expression is shown in Figure 5. cfa-miR-409-3p displayed numerically lower values in advanced stages (III–V) compared with early stages (I–II), whereas cfa-miR-4270 showed the opposite trend, with higher expression at stage I that declined progressively. Cfa-miR-133a exhibited no consistent directional pattern across stages. None of these stage-associated trends reached statistical significance (Kruskal–Wallis, *p* > 0.05 for all), and substantial within-group variability was present throughout, consistent with the exploratory nature of this analysis.

### Correlation analysis

The correlation matrix presented in Figure 3 illustrates the relationship between the expression of microRNAs cfa-miR-409-3p, cfa-miR-4270, and cfa-miR-133a and relevant clinical variables in female dogs with mammary tumors, including lymph node metastasis, tumor stage, spaying status, age, recurrence, and presence of metastasis.

Among the notable findings, a weak positive correlation was observed between the expression of cfa-miR-409-3p and cfa-miR-4270, suggesting that these microRNAs may share common regulatory pathways, although the association is of low intensity.

Additionally, a moderate positive correlation was identified between lymph node metastasis and tumor recurrence, indicating that female dogs with neoplastic dissemination to lymph nodes are more likely to develop disease recurrence.

Furthermore, a moderate positive correlation between recurrence and metastasis was noted, reinforcing the association between tumor progression and cancer recurrence.

No other significant correlations were identified between the analyzed microRNAs and the clinical variables. These findings suggest that, despite the weak association between cfa-miR-409-3p and cfa-miR-4270, these biomarkers may not be directly related to clinical outcomes of mammary neoplasia in female dogs. Further studies are needed to clarify their role in tumor progression.

### Plasma disease free and overall survival

**Figure 6:**
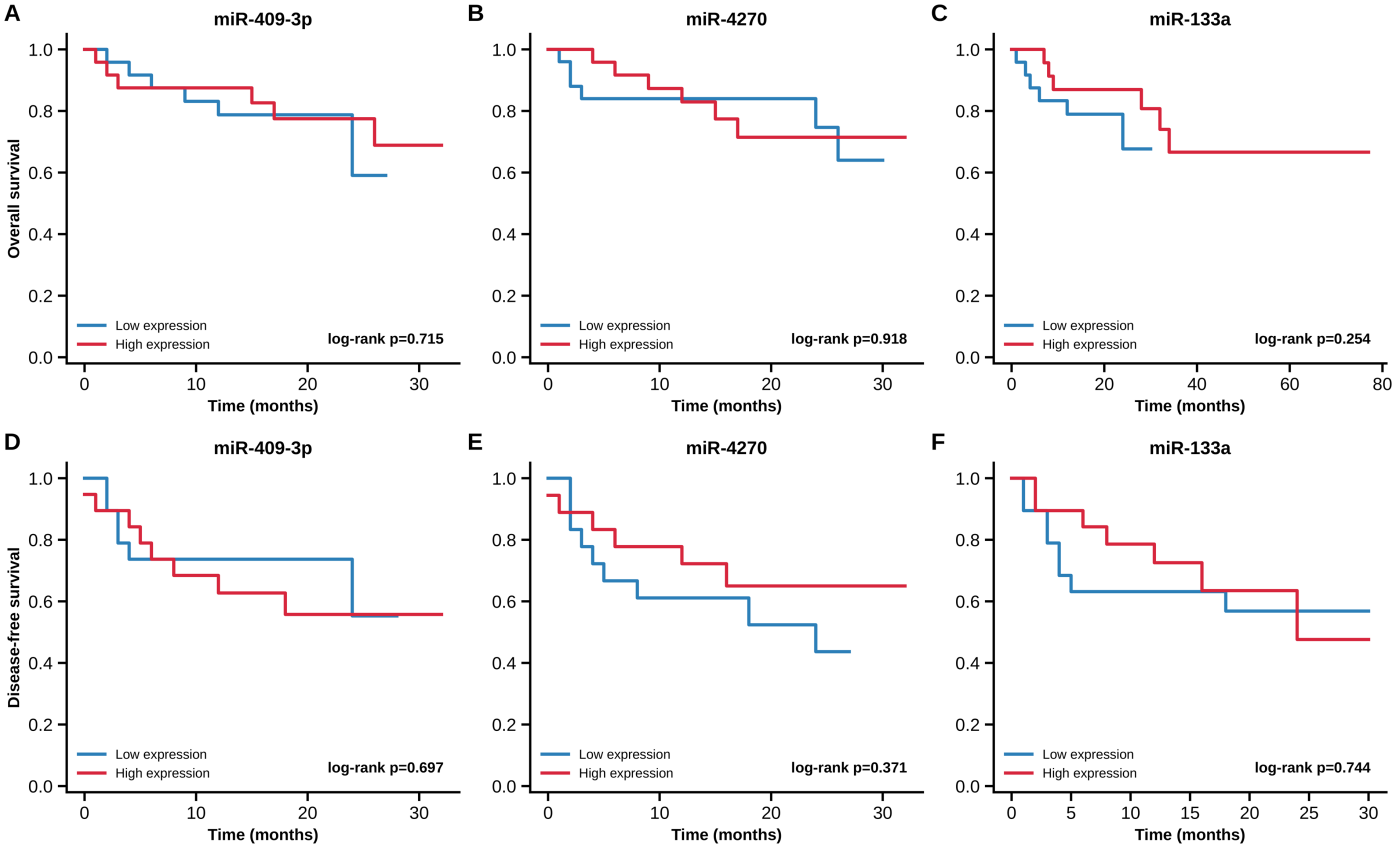
Kaplan–Meier curves by plasma microRNA expression—overall survival **(A–C)** and disease-free survival **(D–F)**; all log-rank *p* > 0.05. Disease-free survival curves associated with microRNA expression. Panels A, B, and C illustrate the disease-free survival (DFS) curves in months, stratified by expression levels (high or low) of three microRNAs: **(A)** cfa-miR-409-3p, **(B)** cfa-miR-4270, and **(C)** cfa-miR-133a. Kaplan–Meier survival curves comparing high- and low-expression groups of cfa-miR-409-3p **(A)**, cfa-miR-4270 **(B)**, and cfa-miR-133a **(C)** in relation to disease-free survival. No statistically significant differences were observed between groups (log-rank test, *p* > 0.05). The blue line represents low microRNA expression, while the red line represents high microRNA expression.

Kaplan–Meier survival curves (Figure 6) were generated to assess the association between plasma expression levels of cfa-miR-409-3p, cfa-miR-4270, and cfa-miR-133a with disease-free interval (DFI) and overall survival (OS). No statistically significant differences were observed between high- and low-expression groups for any of the evaluated microRNAs.

### Genetic ontology

Our gene ontology analysis identified three key pathways influenced by cfa-miR-133a, cfa-miR-409-3p, and cfa-miR-4270, highlighting their intricate roles in tumorigenesis. The involvement of these miRNAs in the JAK–STAT (Supplementary Figure S2), Rap1 (Supplementary Figure S3), and proteoglycan pathways (Supplementary Figure S4) suggest novel regulatory mechanisms that may drive cancer progression.

## Discussion

This exploratory study characterized tissue and plasma expression of five candidate microRNAs across the progression from normal mammary tissue to benign adenoma and invasive carcinoma in female dogs. Three miRNAs—cfa-miR-133a, cfa-miR-409-3p, and cfa-miR-4270—showed statistically significant tissue-level dysregulation that was biologically coherent with progressive malignant transformation, while plasma analysis did not reveal significant differences in any of the clinical comparisons evaluated. Taken together, these findings provide hypothesis-generating evidence regarding the role of specific miRNAs in canine mammary carcinogenesis and highlight the current limitations of plasma-based miRNA quantification in this clinical setting.

CMTs remain the most prevalent malignancy in intact female dogs and represent a clinically and molecularly relevant spontaneous model for human breast cancer (17, 18). The adenoma-to-carcinoma sequence in dogs mirrors the progression from benign epithelial proliferation to invasive carcinoma observed in humans, providing a unique opportunity to study the molecular events associated with malignant transformation (2, 19). While most prior canine miRNA studies have contrasted tumor tissue with normal mammary gland broadly (1, 9), the present study specifically examined expression changes at each stage of progression—normal tissue, adenoma, and carcinoma—an approach that substantially enriches the biological interpretation of the findings.

cfa-miR-133a was significantly downregulated in both adenomas and carcinomas relative to normal mammary tissue, indicating that loss of this miRNA occurs early and is maintained throughout the carcinogenic process. This pattern is consistent with the established tumor suppressor function of the miR-133 family: in human breast cancer, miR-133a suppresses cell proliferation, migration, and invasion through regulation of multiple oncogenic axes including EGFR/PI3K/AKT signaling (20, 21). Notably, Chen et al. (10) previously reported downregulation of the miR-133 family in CMTs by next-generation sequencing; the present study provides independent qPCR-based validation of this finding in a distinct cohort and demonstrates that downregulation begins already at the adenoma stage. The early and sustained loss of cfa-miR-133a across both benign and malignant histotypes suggests it may represent a sensitive, histotype-independent marker of mammary neoplasia in dogs, warranting evaluation in larger prospective studies.

The most biologically compelling finding of this study was the biphasic expression pattern of cfa-miR-409-3p and cfa-miR-4270. Both miRNAs were significantly downregulated in adenomas relative to normal tissue but were subsequently upregulated in carcinomas compared with adenomas. This context-dependent regulation is characteristic of microRNAs with dual functional roles, acting as tumor suppressors in early neoplastic tissue and being functionally reprogrammed during malignant transformation. Such behavior has been extensively documented for miR-409-3p in human cancers: Josson et al. (22) demonstrated that miR-409-3p/−5p promotes tumorigenesis, epithelial-to-mesenchymal transition (EMT), and bone metastasis in prostate cancer, while other studies have identified tumor-suppressive roles for miR-409-3p in osteosarcoma through inhibition of cell proliferation and targeting of oncogenic transcription factors (23). The context-dependent oncomiR vs. tumor suppressor behavior of miR-409-3p likely reflects differences in target gene landscape, co-expressed competing endogenous RNAs, and tumor microenvironment composition across cell types. In CMTs, downregulation in adenomas may represent a loss of early proliferation-restricting function, while subsequent upregulation in carcinomas may promote EMT-associated programs driving invasive behavior. For cfa-miR-4270, while mechanistic data in cancer remain limited, miR-4270 has been identified among differentially upregulated miRNAs in gastric cancer stem cell-like populations (24), suggesting a potential role in cancer cell plasticity. Importantly, Aminisepehr et al. (25) reported dysregulation of miR-4270 in plasma of patients with invasive ductal breast carcinoma, providing the first clinical evidence linking this miRNA to mammary cancer biology and directly supporting its evaluation in canine mammary neoplasia within a comparative oncology framework. The coordinate biphasic regulation of cfa-miR-409-3p and cfa-miR-4270—two miRNAs with overlapping predicted target networks converging on JAK–STAT, Rap1, and proteoglycan signaling pathways (26–29)—raises the possibility of co-regulatory mechanisms during the adenoma-to-carcinoma transition that merit mechanistic investigation using functional *in vitro* models. Notably, the two microRNAs are not genomically co-located: cfa-miR-409 maps to the imprinted DLK1–DIO3 microRNA cluster (canine ortholog of the human 14q32.2 cluster), whereas miR-4270 is annotated on a distinct chromosome (human chromosome 3p21.31; miRBase MI0015878). The strong tissue co-expression (*ρ* = 0.82) is therefore unlikely to reflect co-transcription from a single polycistronic primary transcript and more probably arises from shared transcriptional control by common upstream regulators or from co-induction within EMT-associated programs.

cfa-miR-127-3p showed a progressive numerical increase from normal tissue through adenoma to carcinoma, though without reaching statistical significance, possibly reflecting insufficient statistical power in this pilot cohort. In human breast cancer, miR-127 has been described as a tumor suppressor targeting BCL6 and other oncoproteins, and its overexpression has been associated with inhibition of cancer cell growth (30). A statistically significant upward trend in CMT tissue would be paradoxical from a tumor suppressor perspective; however, context-dependent oncomiR roles similar to those described for miR-409-3p cannot be excluded. Cfa-miR-652 did not demonstrate significant alterations in either tissue or plasma samples. While miR-652 has been reported as part of circulating biomarker panels in human breast cancer (31, 32), its stable expression across CMT histotypes in the present study suggests it may not have a primary regulatory role in canine mammary carcinogenesis, or that relevant dysregulation occurs at stages not captured in this cohort.

Despite tissue-level dysregulation of three microRNAs, no statistically significant differences were observed in plasma across any of the clinical comparisons evaluated, including carcinoma vs. healthy controls, metastatic vs. non-metastatic disease, tumor stage, reproductive status, and survival outcomes. These negative findings are biologically informative and consistent with accumulating evidence that tissue-level miRNA dysregulation does not necessarily translate into proportional changes in circulating plasma concentrations (15). The biological relationship between intratumoral miRNA expression and circulating levels is complex and remains incompletely characterized: factors such as exosomal packaging efficiency, tumor vascularity, and plasma clearance kinetics may all contribute to tissue-plasma expression discordance (14, 15). Additionally, the use of different quantification platforms for tissue and plasma analysis reflects the exploratory, multi-compartment design of this study and underscores the value of adopting harmonized cross-platform protocols in future investigations.

The use of poly(A) tailing-based SYBR Green RT-qPCR for tissue miRNA quantification in the present study represents a technically validated approach to mature miRNA detection. This method, first described by Shi and Chiang (33), polyadenylates total RNA using poly(A) polymerase, enabling reverse transcription with a universal oligo(dT) adapter primer and subsequent PCR amplification with miRNA-specific forward primers and a universal reverse primer. The approach has been shown to discriminate mature miRNAs differing by a single nucleotide and is sensitive to as little as 100 pg. of total RNA (33). The primer design in this study—evidenced by the long universal reverse primer in Supplementary Table S2—is consistent with this poly(A) tailing methodology, confirming that mature miRNA sequences were specifically targeted. For future studies, harmonization of tissue and plasma quantification methods using a single validated platform would strengthen the translational conclusions.

Gene ontology analysis predicted that cfa-miR-133a, cfa-miR-409-3p, and cfa-miR-4270 share enriched target gene networks converging on JAK–STAT, Rap1, and proteoglycan biosynthesis signaling pathways (26–29). These pathways govern central aspects of tumor biology including cytokine-driven proliferation, cell–cell and cell-matrix adhesion, stromal remodeling, and metastatic dissemination. The convergence of multiple dysregulated miRNAs on overlapping pathway networks may amplify their combined regulatory impact on CMT progression, and warrants network-level functional validation in canine mammary epithelial cell lines and organoid models. The biological conservation of these pathway interactions between dogs and humans further supports the comparative oncology value of CMTs as a platform for miRNA mechanistic studies. Importantly, these pathway analyses remain descriptive in the present study: the predicted targets were not experimentally validated, and the links between specific dysregulated microRNAs, their downstream effectors, and the observed tumour phenotypes were not formally established. Future work should integrate microRNA expression with target-gene validation and pathway-level modelling—ideally summarised in a pathway-centred schematic linking cfa-miR-133a, cfa-miR-409-3p and cfa-miR-4270 to epithelial-to-mesenchymal transition, proliferation and invasion—to strengthen the mechanistic and translational interpretation of these findings.

As an exploratory pilot study, the present work has boundaries that frame, rather than undermine, its conclusions. Because the analysis was designed to generate hypotheses, expression differences were examined without formal correction for multiple comparisons—although, reassuringly, the three significant tissue microRNAs remained significant after Benjamini–Hochberg adjustment (Supplementary Table S3)—and the findings are therefore best regarded as preliminary until confirmed in larger, prospectively stratified cohorts with multivariable adjustment, particularly given the modest age and reproductive-status differences between the groups. The circulating arm was constrained chiefly by the small healthy-control group, so the absence of significant plasma differences is most appropriately interpreted as no detectable signal in an underpowered cohort rather than as evidence against circulating biomarker potential; the same caution applies to the survival analyses, which were based on few events and on median dichotomization of expression. Our experience also points to methodological refinements for future work: complementing U6—whose stability differs between tissue and plasma—with spike-in (e.g., cel-miR-39) or multi-reference normalization (34), harmonizing the RT-qPCR chemistry used across the two compartments, and adopting quantitative rather than visual assessment of hemolysis. Finally, functional validation through target-gene and pathway-level assays in canine mammary models will be needed to establish whether these microRNAs causally contribute to malignant transformation.

In this exploratory study, tissue microRNA analysis identified biologically coherent dysregulation patterns across the canine mammary adenoma-to-carcinoma sequence. Cfa-miR-133a was consistently downregulated from the earliest neoplastic stage, consistent with a conserved tumor suppressor role, while cfa-miR-409-3p and cfa-miR-4270 displayed a biphasic expression pattern that distinguishes adenoma from carcinoma and suggests context-dependent reprogramming during malignant transformation. These tissue findings align with known roles of human orthologs in breast and other cancers, supporting the translational relevance of the CMT model for miRNA biomarker research. Plasma microRNA expression did not differ significantly between groups; however, circulating microRNA detection remains technically and biologically challenging in this context, and larger, standardized, adequately powered studies employing harmonized quantification platforms and validated multi-gene reference panels will be required to establish its feasibility. Together, the tissue and plasma data generated here provide a structured hypothesis-generating foundation for future investigations into the diagnostic and prognostic potential of microRNAs in canine mammary oncology.

## Data Availability

The original contributions presented in the study are included in the article/Supplementary material, further inquiries can be directed to the corresponding author.
