# Supplementary material for: Biphasic tissue expression of cfa-miR-409-3p and cfa-miR-4270 during malignant transformation in canine mammary tumors: an exploratory study
Source: Front Vet Sci. 2026 Jun 23;13:1861662. doi: 10.3389/fvets.2026.1861662 (PMC13338724; doi:10.3389/fvets.2026.1861662)
Supplement: Supplementary file 1 [file Supplementary_file_1.zip › Supplementary file 1/Image_1.pdf]

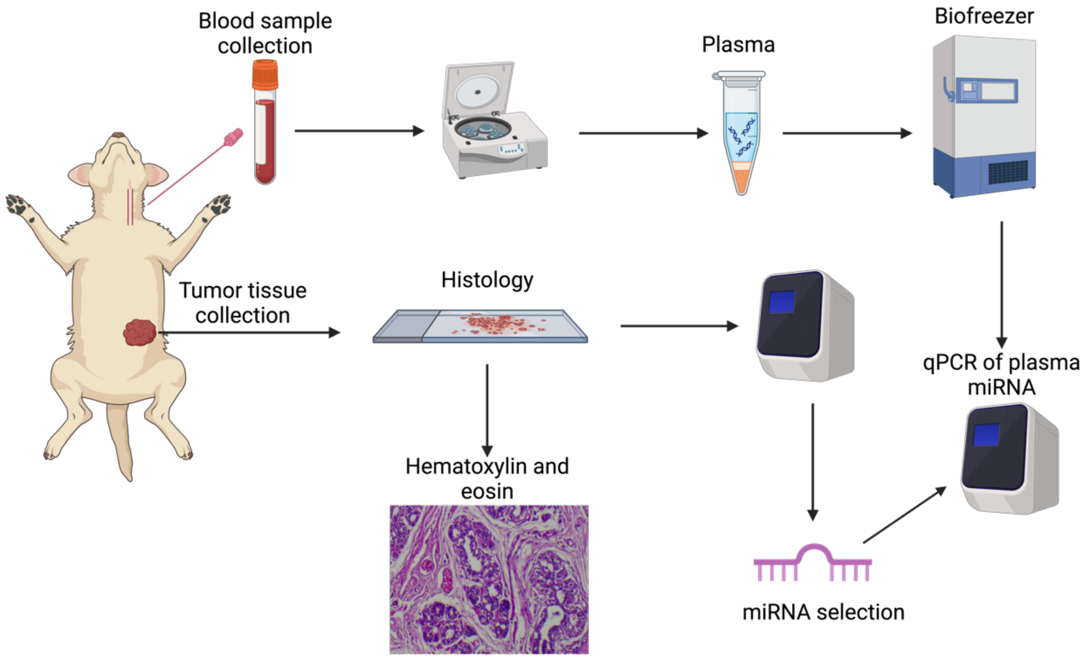

Supplementary Figure 1- Workflow for sample processing in the analysis of microRNA expression in bitches with mammary tumors. Peripheral blood samples were collected, centrifuged for plasma separation, and stored for subsequent RNA extraction and quantification by real-time PCR. Simultaneously, tumor fragments were processed for histopathological analysis and RNA extraction to assess differential microRNA expression.

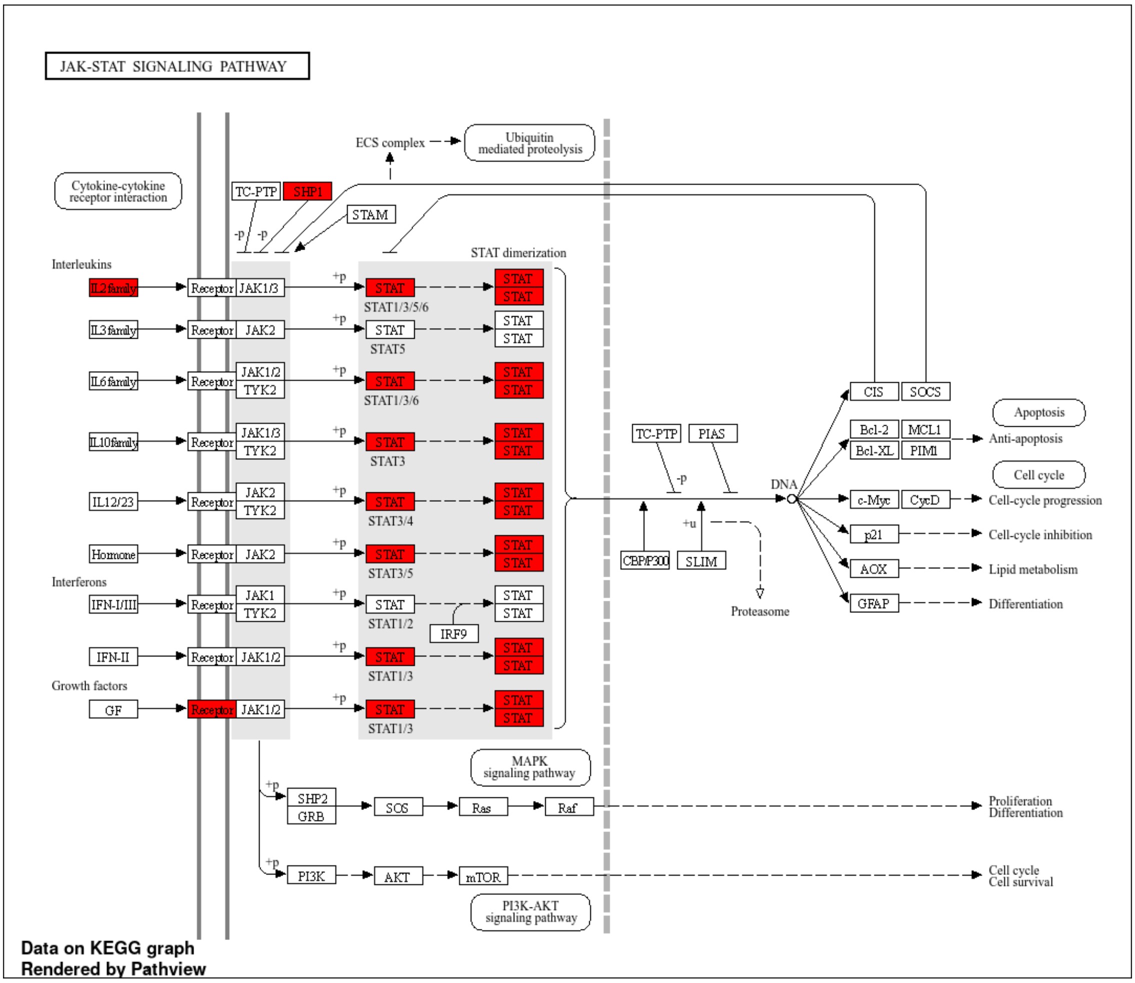

Supplementary Figure 2 - Regulation of the JAK-STAT pathway by miRNAs cfa-miR-133a, cfa-miR-409-3p, and cfa-miR-4270. Gene ontology analysis revealed that these miRNAs regulate key genes in this pathway, including JAK1, JAK2, STAT3, and STAT5. The modulation of these genes may directly impact cell proliferation, differentiation, and immune response, contributing to tumorigenic processes in canine mammary neoplasms. Genes highlighted in red are those directly regulated by the miRNAs.

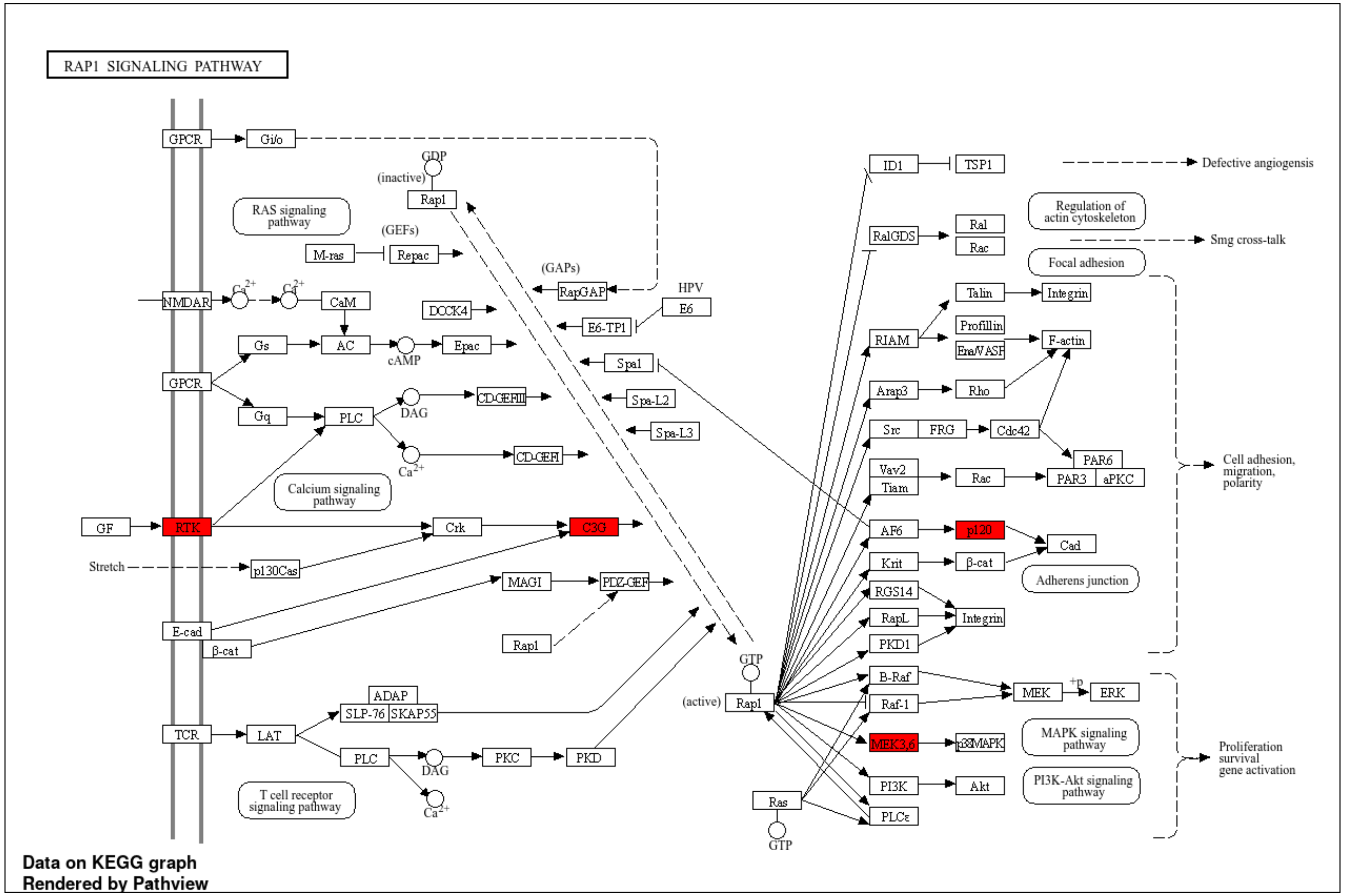

Supplementary Figure 3 - Modulation of the Rap1 pathway by miRNAs cfa-miR-133a, cfa-miR-409-3p, and cfa-miR-4270. The Rap1 pathway, which is involved in cell adhesion and migration, was significantly affected by the analyzed miRNAs. The regulated genes include RTK, C3G, and MEK3/6, suggesting a potential impact on tumor progression and the metastatic capacity of neoplastic cells. The regulation of this pathway may promote cytoskeletal alterations and MAPK signaling, enhancing cell survival and invasiveness.

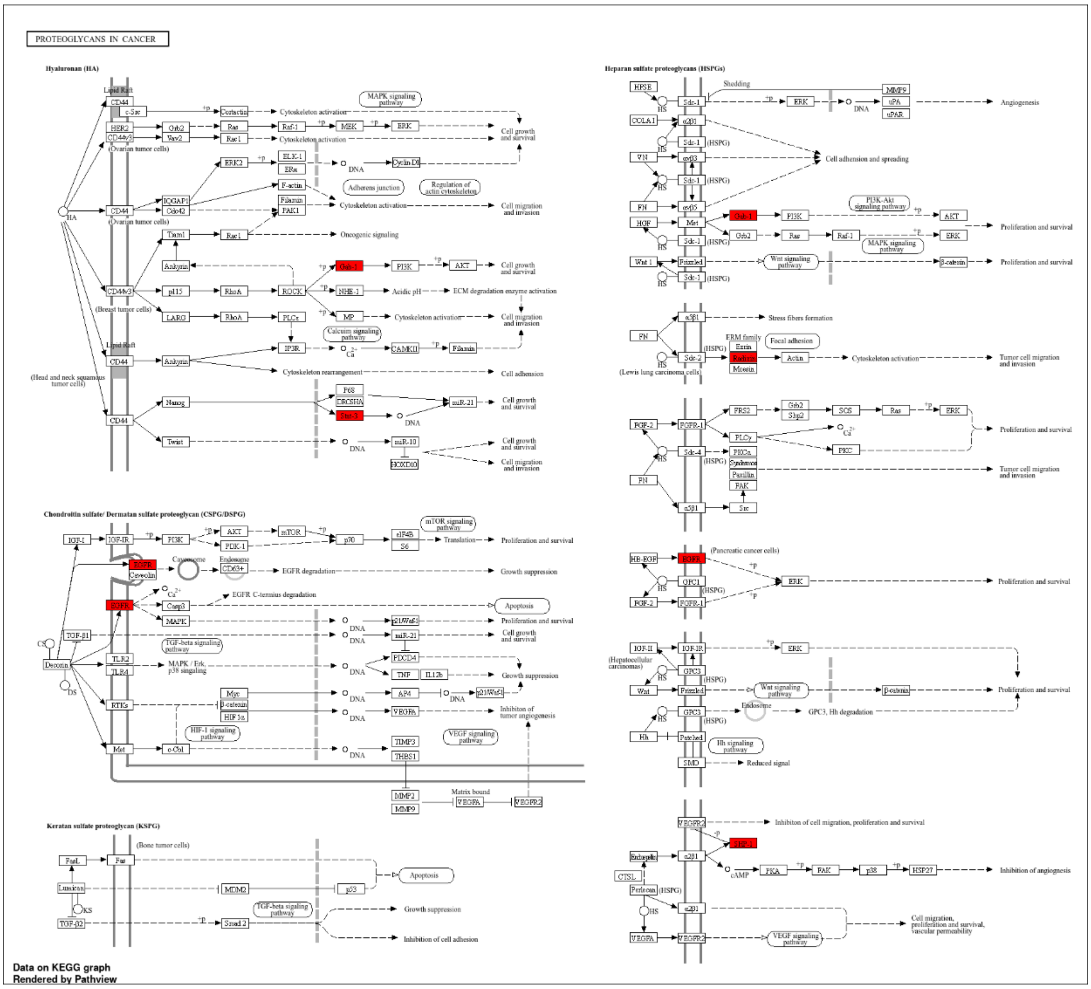

Supplementary Figure 4 - Influence of miRNAs cfa-miR-133a, cfa-miR-409-3p, and cfa-miR-4270 on the proteoglycan pathway in cancer. The studied miRNAs were found to regulate genes such as HSPG2, SDC1, and BCL2, which play central roles in modulating the tumor microenvironment. The repression of BCL2 suggests a potential pro-apoptotic effect, while HSPG2 and SDC1 may enhance cell-extracellular matrix interactions, promoting invasion and cell survival. The regulation of these genes underscores the significance of these pathways in canine mammary cancer progression.
