# Supplementary material for: Biphasic tissue expression of cfa-miR-409-3p and cfa-miR-4270 during malignant transformation in canine mammary tumors: an exploratory study
Source: Front Vet Sci. 2026 Jun 23;13:1861662. doi: 10.3389/fvets.2026.1861662 (PMC13338724; doi:10.3389/fvets.2026.1861662)
Supplement: Supplementary file 1 [file Supplementary_file_1.zip › Supplementary file 1/Table S1 and S2.DOCX]

**Supplementary Table 1 – Candidate microRNAs selected for evaluation in canine mammary tumors. microRNA identifiers follow miRBase nomenclature (Canis familiaris, cfa-). CMTs, canine mammary tumors; EMT, epithelial-to-mesenchymal transition.**

| **microRNA** | **Evidence source (reference)** | **Rationale for inclusion** | **miRBase accession** |
| --- | --- | --- | --- |
| **cfa-miR-133a** | Hua et al., 2021; Barartabar et al., 2023; Chen et al., 2022 | Reported as a tumor suppressor in breast and other cancers; regulates apoptosis, proliferation, and  chemotherapy sensitivity | MI0010326 |
| **cfa-miR-127-3p** | Cuk et al., 2013 | Validated as circulating biomarker in breast cancer plasma; detected in stages I and II as part of a seven-miRNA diagnostic panel | MI0008132 |
| **cfa-miR-652** | Lagendijk et al., 2018; Cuk et al., 2013 | Circulating biomarker in breast cancer; included in diagnostic miRNA panels | MI0008167 |
| **cfa-miR-409-3p** | Cuk et al., 2013; Josson et al., 2014 | Validated in breast cancer circulating miRNA panel; promotes EMT and metastasis in human cancers; biphasic expression pattern in CMTs | MI0008147 |
| **cfa-miR-4270** | Aminisepehr et al., 2018 | Reported to be altered in plasma of patients with invasive ductal carcinoma | MI0015878 (hsa-miR-4270; no Canis familiaris entry) |

**Supplementary Table 2 – qPCR primer sequences for the five candidate microRNAs (cfa-miR-127-3p, cfa-miR-652, cfa-miR-409-3p, cfa-miR-133a, cfa-miR-4270). For each microRNA, the table lists the microRNA-specific forward primer and the universal reverse primer used for SYBR Green qPCR following poly(A) tailing-based reverse transcription. This single table provides the complete primer information for all five candidates.**

| microRNA | Forward primer (5’–3’) | Reverse primer (5’–3’) |
| --- | --- | --- |
| cfa-miR-127-3p | CGGCGGTCGGATCCGTCTGAGC | GTCGTATCCAGTGCAGGGTCCGAGGTATTCGCACTGGATACGACAGCCAA |
| cfa-miR-652 | CGGCGGAATGGCGCCACTAGGG | GTCGTATCCAGTGCAGGGTCCGAGGTATTCGCACTGGATACGACGCACAA |
| cfa-miR-409-3p | CGGCGGAATGTTGCTCGTTGAACCCCT | GTCGTATCCAGTGCAGGGTCCGAGGTATTCGCACTGGATACGACAGGGGT |
| cfa-miR-133a | CGGCGGTTGGTCCCCTTCAACC | GTCGTATCCAGTGCAGGGTCCGAGGTATTCGCACTGGATACGACACAGCT |
| cfa-miR-4270 | CGGCGGTCAGGGAGTCAGGG | GTCGTATCCAGTGCAGGGTCCGAGGTATTCGCACTGGATACGACGCCCTC |
