# Supplementary material for: Biphasic tissue expression of cfa-miR-409-3p and cfa-miR-4270 during malignant transformation in canine mammary tumors: an exploratory study
Source: Front Vet Sci. 2026 Jun 23;13:1861662. doi: 10.3389/fvets.2026.1861662 (PMC13338724; doi:10.3389/fvets.2026.1861662)
Supplement: Supplementary file 1 [file Supplementary_file_1.zip › Supplementary file 1/Table S3.DOCX]

**Supplementary Table 3. Tissue microRNA expression: descriptive statistics, group comparisons, effect sizes and fold-changes.**

| **miRNA** | **n (N/A/C)** | **Normal median[IQR]** | **Adenoma median[IQR]** | **Carcinoma median[IQR]** | **KW p** | **KW ε²** | **Dunn A vs N (BH)** | **Dunn C vs A (BH)** | **Dunn C vs N (BH)** | **log2FC A/N (95%CI)** | **log2FC C/A (95%CI)** | **KW BH q** |
| --- | --- | --- | --- | --- | --- | --- | --- | --- | --- | --- | --- | --- |
| cfa-miR-133a | 10/11/28 | 1.217 [0.534–2.928] | 0.038 [0.015–0.059] | 0.034 [0.019–0.083] | <0.001 | 0.384 | <0.001 | 0.694 | <0.001 | -5.01 (-7.32,-3.30) | -0.16 (-1.18,+1.68) | <0.001 |
| cfa-miR-409-3p | 9/12/30 | 5.822 [4.872–8.630] | 1.289 [1.000–1.475] | 1.571 [1.282–3.122] | <0.001 | 0.311 | <0.001 | 0.054 | 0.004 | -2.17 (-3.06,-1.76) | +0.28 (-0.01,+0.78) | <0.001 |
| cfa-miR-4270 | 10/13/31 | 1.292 [1.094–1.865] | 0.161 [0.118–0.248] | 0.302 [0.214–0.877] | <0.001 | 0.361 | <0.001 | 0.008 | 0.008 | -3.00 (-3.84,-2.32) | +0.91 (+0.26,+1.65) | <0.001 |
| cfa-miR-127-3p | 10/10/29 | 1.204 [0.488–1.444] | 1.608 [1.111–2.106] | 1.095 [0.292–3.034] | 0.526 | -0.016 | 0.555 | 0.555 | 0.555 | +0.42 (-0.28,+1.93) | -0.55 (-1.69,+0.90) | 0.658 |
| cfa-miR-652 | 10/13/31 | 1.102 [0.729–1.345] | 1.021 [0.923–1.264] | 1.087 [0.699–1.418] | 0.886 | -0.034 | 0.916 | 0.916 | 0.916 | -0.11 (-0.45,+0.64) | +0.09 (-0.31,+0.42) | 0.886 |

*N/A/C, Normal/Adenoma/Carcinoma; IQR, interquartile range; KW, Kruskal–Wallis; ε², epsilon-squared effect size; Dunn comparisons are Benjamini–Hochberg (BH)-adjusted; log2FC, log2 fold-change with bootstrap 95% confidence interval; q, BH false-discovery-rate q-value across the five Kruskal–Wallis tests.*
